# Human dental pulp stem cells (hDPSCs) promote the lipofibroblast transition in the early stage of a fibro-inflammatory process

**DOI:** 10.3389/fcell.2023.1196023

**Published:** 2023-05-03

**Authors:** Alessandra Pisciotta, Rosanna Di Tinco, Giulia Bertani, Giulia Orlandi, Laura Bertoni, Elisa Pignatti, Monia Orciani, Paola Sena, Jessika Bertacchini, Carlo Salvarani, Gianluca Carnevale

**Affiliations:** ^1^ Department of Surgery, Medicine, Dentistry and Morphological Sciences with Interest in Transplant, Oncology and Regenerative Medicine, University of Modena and Reggio Emilia, Modena, Italy; ^2^ Department of Clinical and Molecular Sciences, Polytechnic University of Marche, Ancona, Italy; ^3^ Unit of Rheumatology, Azienda Unità Sanitaria Locale-IRCCS, Reggio Emilia, Italy

**Keywords:** human dental pulp stem cells, pericytes, immunomodulation, fibrosis, TGF-β1

## Abstract

**Introduction:** In autoimmune diseases, particularly in systemic sclerosis and chronic periaortitis, a strict correlation between chronic inflammation and fibrosis exists. Since the currently used drugs prove mostly effective in suppressing inflammation, a better comprehension of the molecular mechanisms exerted by cell types implicated in fibro-inflammation is needed to develop novel therapeutic strategies. Mesenchymal stromal/stem cells (MSCs) are being matter of deep investigation to unveil their role in the evolution of fibrogenetic process. Several findings pointed out the controversial implication of MSCs in these events, with reports lining at a beneficial effect exerted by external MSCs and others highlighting a direct contribution of resident MSCs in fibrosis progression. Human dental pulp stem cells (hDPSCs) have demonstrated to hold promise as potential therapeutic tools due to their immunomodulatory properties, which strongly support their contribution to tissue regeneration.

**Methods:** Our present study evaluated hDPSCs response to a fibro-inflammatory microenvironment, mimicked in vitro by a transwell co-culture system with human dermal fibroblasts, at early and late culture passages, in presence of TGF-β1, a master promoter of fibrogenesis.

**Results and Discussion:** We observed that hDPSCs, exposed to acute fibro-inflammatory stimuli, promote a myofibroblast-to-lipofibroblast transition, likely based on BMP2 dependent pathways. Conversely, when a chronic fibro-inflammatory microenvironment is generated, hDPSCs reduce their anti-fibrotic effect and acquire a pro-fibrotic phenotype. These data provide the basis for further investigations on the response of hDPSCs to varying fibro-inflammatory conditions.

## 1 Introduction

Inflammation is a physiological defense mechanism against different stimuli. When inflammation is chronically perpetrated, a progressive fibrosis with an excessive accumulation of extracellular matrix (ECM) components may occur. This fibro-inflammatory process leads to disrupted tissue function and organ damage (Medzhitov, 2008; Jeljeli et al., 2019) as well demonstrated by the strict relationship between inflammation and fibrosis characterizing some chronic autoimmune diseases. Interleukin-6 (IL-6) plays a key pathogenic role in experimental models of systemic sclerosis and graft-versus-host diseases (O’Reilly et al., 2012). This cytokine is one of the most important drivers of inflammatory response, and there is evidence that these conditions and retroperitoneal fibrosis (i.e., chronic periaortitis), all hallmarked by an intense fibrosing response, respond to IL-6 blockade with tocilizumab (Vaglio et al., 2013; Khanna et al., 2020).

However, there has been a limited availability of effective therapies, all prevalently targeting inflammation. Indeed, corticosteroids are the most used drugs that suppress the production of inflammatory cytokines during the acute phase. In parallel, in steroid-refractory patients or to spare corticosteroids, a therapy based on immunosuppressive or biological agents is usually associated with corticosteroid therapy (Vaglio et al., 2006). A better understanding of the main molecular mechanisms regulating the fibro-inflammatory response in these diseases is important for the development of novel treatment strategies that will be able to spare or replace corticosteroids for reducing serious corticosteroid side effects.

Different cell types that activate molecular signaling pathways in response to fibrosis-related factors are involved in fibro-inflammatory processes. In this regard, it is well known that transforming growth factor-beta (TGF-β) is a central pro-fibrotic mediator and a master regulator that induces a mesenchymal transition in a variety of cells, through TGF-β/Smad- and non-Smad-mediated signaling pathways (Meng et al., 2016).

The fibrogenic role of mesenchymal stromal cells (MSCs), derived from different sources, such as skeletal muscle, adipose tissue, placenta, umbilical cord, liver, and dental pulp, is highly debated. Several findings have shown that MSCs can behave as primary fibrosis-forming cells as supported by studies of fibrogenesis in liver, kidneys, lungs, spinal cord, and systemic sclerosis (LeBleu et al., 2013; El Agha et al., 2017a; Kuppe et al., 2021). Recently, perivascular GLI1^+^ MSC-like cells were identified as a major cellular origin of organ fibrosis and thus might represent a therapeutic target to prevent fibrotic organ dysfunction (Schneider et al., 2017).

Conversely, MSCs were also demonstrated to be involved in the reduction of fibro-inflammatory processes (Alfaifi et al., 2018; Peltzer et al., 2018; Li et al., 2021; Qin et al., 2023). As a matter of fact, it has been documented that MSCs modulate macrophage phenotypes by reducing the proportion of the pro-fibrotic cell phenotype (M2) and exerting anti-fibrotic effects (Willis et al., 2018; Luo et al., 2019). Furthermore, they directly counteract the fibrotic process by modulating the ratio of metalloproteinases/metalloproteinase tissue inhibitors, thereby reducing the content of collagen fibers and inhibiting lung remodeling (Xu et al., 2017; Chu et al., 2019).

Among different MSC types, human dental pulp stem cells (hDPSCs) have been demonstrated to display pericyte-like features, to own a wide differentiation potential *in vitro* and to contribute to regenerating processes and to the maintenance of tissue homeostasis through direct histointegration, immunomodulatory abilities, and promotion of neoangiogenesis (Janebodin et al., 2013; Nuti et al., 2016; Martinez-Sarrà et al., 2017; Paganelli et al., 2021). Recently, our research group has demonstrated that hDPSCs are able to modulate the immune response by activating different pathways, i.e., PD1/PD-L1 and Fas/FasL, and that the surrounding microenvironment may affect their immunomodulatory/inflammatory phenotype (Di Tinco et al., 2021; Bertani et al., 2023). However, little is known about the role of hDPSCs in fibrosis processes. Based on these considerations, the aim of the present study was to evaluate the role of hDPSCs in an *in vitro* model of fibro-inflammation mimicked by a co-culture system with fibroblasts exposed to TGF-β1.

## 2 Materials and methods

### 2.1 Fibroblast culture and stimulation with TGF-β1

Human dermal fibroblasts (hDFs; C0135C, Thermo Fisher Scientific, Waltham, MA, United States) were cultured in Eagle’s minimum essential medium alpha modification (α-MEM; Sigma-Aldrich, St. Louis, MO, United States) supplemented with 10% heat-inactivated fetal bovine serum (FBS; Euroclone, Milan, Italy), 2 mM L-glutamine, 100 U/mL penicillin, and 100 μg/mL streptomycin (Sigma-Aldrich) up to the third passage. hDFs cultured upon passage 3 (P3; early culture passage) and passage 12 (P12; late culture passage) were then seeded at a cell density of 3,000 cells/cm^2^. When reaching 60% confluence, hDFs, at both culture passages P3 and P12, were stimulated with the addition of human recombinant TGF-β1 (10 ng/mL; Abbkine, Wuhan, China) for 3 and 7 days, to induce the fibroblast-to-myofibroblast conversion.

To evaluate the effects of BMP2 on TGF-β1-pre-stimulated hDFs (P3), 10 ng/mL rhTGF-β1 and 50 ng/mL rhBMP2 (OriGene, Rockville, MD, United States) were added, according to previous findings (Kheirollahi et al., 2019) for further 4 days.

### 2.2 Isolation and immune selection of human dental pulp stem cells

The study was performed in accordance with the recommendations of Comitato Etico Provinciale—Azienda Ospedaliero-Universitaria di Modena (Modena, Italy), which provided the approval of the protocol (ref. number 3299/CE, September 2017).

The dental pulp was harvested from third molars of adult subjects (*n* = 3; 18–35 years), after obtaining their written informed consent, in compliance with the Declaration of Helsinki. The dental pulp was digested in α-MEM containing 3 mg/mL type I collagenase plus 4 mg/mL dispase (all from Sigma-Aldrich) and then was filtered onto 100-μm Falcon Cell Strainers to obtain a cell suspension. Cell suspension was then plated in 25-cm^2^ culture flasks and expanded in a culture medium at 37°C and 5% CO_2_. Following cell expansion, human dental pulp cells underwent magnetic cell sorting, using the MACS^®^ separation kit, against the stemness surface markers STRO-1 and c-Kit, which allow to obtain a purer stem cell population within human dental pulp. hDPSCs were sequentially immune-selected as previously described (Di Tinco et al., 2021). Mouse IgM anti-STRO-1 and rabbit IgG anti-c-Kit primary antibodies (Santa Cruz Biotechnology, Dallas, TX, United States) were used and subsequently revealed by magnetically labeled anti-mouse IgM and anti-rabbit IgG secondary antibodies (Miltenyi Biotec, Bergisch Gladbach, Germany). The obtained STRO-1^+^/c-Kit^+^ hDPSCs were expanded and used for any experimental evaluation at passage 3.

### 2.3 Establishment of co-cultures between hDPSCs and TGF-β1-treated hDFs

The transwell co-culture system between hDFs (P3 and P12), previously stimulated with TGF-β1, and hDPSCs (seeded in 1:1 ratio) was set up in six-well insert plates. Briefly, hDFs were first seeded on the bottom of the culture plate at 3,000 cells/cm^2^; then, 24 h later, cells were stimulated with 10 ng/mL rhTGF-β1 (Abbkine) and cultured under these conditions for 3 and 7 days. The TGF-β1 stimulus was administered twice a week. After 3 and 7 days of culturing hDFs with TGF-β1 stimulation, hDPSCs were seeded on transwell inserts, i.e., 0.4 µm polycarbonate membranes (Corning, New York, NY, United States) and maintained in co-culture for 4 days with TGF-β1-treated hDFs (3 and 7 days), still under TGF-β1 exposure. hDPSCs and hDFs cultured alone and without exposure to rhTGF-β1 were used as controls.

### 2.4 Real-time PCR analyses

Real-time PCR analyses were carried out to evaluate the mRNA levels of ACTA2, FN1, COL1A1, HDAC4, TNC, SPARC, BMP2, BMP4, and BMP6. hDFs and hDPSCs were homogenized, and total RNA was extracted and purified using the PureLink RNA columns (Thermo Fisher Scientific). cDNA synthesis was performed using Maxima First Strand cDNA Synthesis Kit with DNase I treatment (Thermo Fisher Scientific). Quantitative real-time PCRs were performed using SYBR Green Master Mix (Bio-Rad) on the CFX Connect Real-time PCR instrument (Bio-Rad, Hercules, CA, United States) with the oligonucleotides. The oligonucleotides used in this study are listed in Table 1 (all obtained from Sigma-Aldrich).

**TABLE 1:** Oligonucleotide sequences used in real-time PCR analyses.

Target gene	Forward sequence	Reverse sequence
hRPLP0	TAC​ACC​TTC​CCA​CTT​GCT​GA	CCA​TAT​CCT​CGT​CCG​ACT​CC
HDAC4	ACA​AGG​AGA​AGG​GCA​AAG​AG	GCG​TTT​TCC​CGT​ACC​AGT​AG
FN1	CCG​CCG​AAT​GTA​GGA​CAA​GA	CGG​GAA​TCT​TCT​CTG​TCA​GCC
TNC	CAC​TAC​ACA​GCC​AAG​ATC​CAG	TCG​TGT​CTC​CAT​TCA​GCA​TTG
ACTA2	AAT​GCA​GAA​GGA​GAT​CAC​GG	TCC​TGT​TTG​CTG​ATC​CAC​ATC
SPARC	CAA​GAA​GCC​CTG​CCT​GAT​GA	TGG​GAG​AGG​TAC​CCG​TCA​AT
COL1A1	CCC​CTG​GAA​AGA​ATG​GAG​ATG	TCC​AAA​CCA​CTG​AAA​CCT​CTG
hBMP2	CTG​CGG​TCT​CCT​AAA​GGT​CG	AGC​AGC​AAC​GCT​AGA​AGA​CA
hBMP4	CTG​CAA​CCG​TTC​AGA​GGT​CC	ACG​GAA​TGG​CTC​CAT​AGG​TC
hBMP6	TTC​CCA​TCC​TTT​CTG​CGA​GC	GGC​GAG​GAT​CTT​GCT​TTC​CG

Relative quantification was calculated from the ratio of the cycle number (Ct) at which the signal crossed a threshold set within the logarithmic phase of the given gene to that of the reference hRPLP0. Mean values of the duplicate results of three independent experiments for each sample were used as individual data for 2^−ΔΔCt^ statistical analysis.

### 2.5 Confocal immunofluorescence analyses

Immunofluorescence analyses were performed on hDPSCs and fibroblasts as previously described (Pisciotta et al., 2022). Cells were fixed in 4% paraformaldehyde in pH 7.4 phosphate-buffered saline (PBS) for 20 min and washed in PBS. Then, where needed, cell permeabilization was performed by using 0.1% Triton X-100 (Sigma-Aldrich) in PBS for 5 min. Samples were washed thrice with PBS and blocked with 3% BSA in PBS for 30 min at room temperature and then were incubated with the primary antibodies [mouse anti-α-SMA (Invitrogen, Waltham, MA, United States), mouse anti-Coll-I (Abcam, Cambridge, UK), mouse anti-fibronectin (Invitrogen), rabbit anti-PPARγ (Cell Signaling Technology, Danvers, MA, United States), rabbit anti-GLI1 (Invitrogen), and rabbit anti-PDGFRβ (Cell Signaling Technology)] diluted 1:50 in PBS containing 3% BSA for 1 h at room temperature. After rinsing with PBS containing 3% BSA, cells were incubated for 1 h at room temperature with secondary antibodies diluted 1:200 in PBS containing 3% BSA (goat anti-mouse Alexa 488, goat anti-rabbit Alexa 488, and donkey anti-mouse Alexa 546; Invitrogen). Finally, after rinsing with PBS, cell nuclei were stained with 1 μg/mL DAPI in PBS for 7 min, and then samples were mounted with Fluoromount anti-fading medium (Invitrogen). Samples were observed using a Nikon A1 confocal laser scanning microscope (Nikon, Minato, Tokyo, Japan). The confocal serial sections were processed using ImageJ software to obtain three-dimensional projections, and image rendering was performed using Adobe Photoshop software (Pisciotta et al., 2022).

### 2.6 Western blot analyses

Whole-cell lysates were obtained as previously described (Di Tinco et al., 2021). A measure of 30 µg of protein extract for each sample was quantified by Bradford protein assay (Bio-Rad), and separation was performed by SDS-polyacrylamide gel electrophoresis on Mini-PROTEAN^®^ TGX™ Stain-Free Precast gels. Gels were then UV-activated using a ChemiDoc MP Imaging System (Bio-Rad), and proteins were subsequently transferred to 0.2-μm nitrocellulose membranes (Bio-Rad). The membranes were then imaged using the ChemiDoc Imaging System (Bio-Rad) for total protein normalization. Membranes were incubated overnight with the following primary antibodies: mouse anti-α-SMA (Invitrogen), mouse anti-fibronectin (Invitrogen), rabbit anti-PPARγ (Cell Signaling Technology), rabbit anti-PDGFRβ (Cell Signaling Technology), and rabbit anti-GLI1 (Invitrogen), all diluted 1:1,000 in 0.1% TBS-Tween 20 (Sigma-Aldrich) and then were incubated with HRP-conjugated anti-rabbit and anti-mouse secondary antibodies (1:3,000; Thermo Fisher Scientific) for 30 min at room temperature. The membranes were visualized using a ChemiDoc Imaging System (Bio-Rad). Finally, the relative expression levels of each evaluated marker were then obtained by normalizing the density of the protein bands to the corresponding stain-free blot image using Image Lab software (Version 6.1, Bio-Rad).

### 2.7 Statistical analysis

All the experiments were performed in triplicate. Data were expressed as mean ± standard deviation (SD). One-way ANOVA followed by the Newman–Keuls *post hoc* test was performed to analyze differences among three or more experimental groups. Differences between co-culture and control groups were analyzed by one-way ANOVA followed by Dunnett’s *post hoc* test (GraphPad Prism software version 8 Inc., San Diego, CA, United States). In any case, statistical significance was set at *p*-values < 0.05.

## 3 Results

### 3.1 Fibroblast-to-myofibroblast conversion is modulated by transwell co-culture with hDPSCs

**FIGURE 1:**
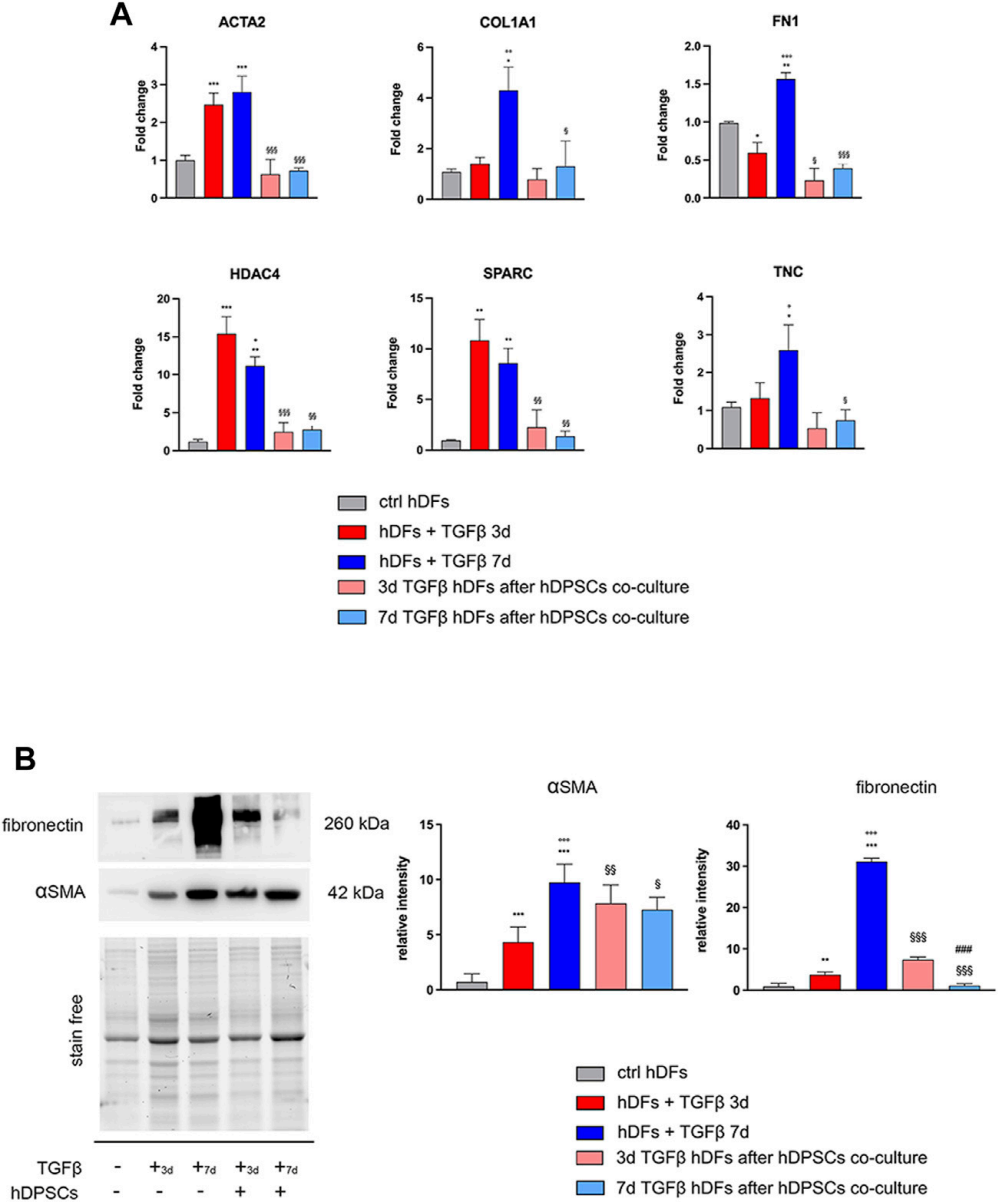
Effects of TGF-β1 stimulation and hDPSC co-culture on human dermal fibroblasts. **(A)** The expression of ACTA2, COL1A1, FN1, HDAC4, SPARC, and TNC was evaluated through real-time PCR analyses on hDFs (P3) stimulated with TGF-β1, cultured alone and after co-culture with hDPSCs. Data are expressed as mean ± SD and analyzed by one-way ANOVA followed by the Newman–Keuls *post hoc* test. *p < 0.05, **p < 0.01 vs ctrl hDFs; °*p* < 0.05, °°*p* < 0.01, °°°*p* < 0.001 vs hDFs + TGFβ 3 days; ^§^ *p* < 0.05, ^§§^ *p* < 0.01, ^§§§^ *p* < 0.001 vs hDFs + TGFβ 3 days and hDFs + TGFβ 7 days. **(B)** Western blot analyses performed on hDFs pre-stimulated with TGF-β, for 3 and 7 days, alone and after hDPSC co-culture. Histograms show that hDFs were induced toward the expression of pro-fibrotic markers α-SMA and fibronectin after TGF-β1 stimulation and that after hDPSC co-culture, the expression of these markers was modulated. Data are expressed as mean ± SD and analyzed by one-way ANOVA followed by the Newman–Keuls *post hoc* test. **p < 0.01, ***p < 0.001 vs ctrl hDFs; °°°*p* < 0.001 vs hDFs + TGFβ 3 days; ^§^ *p* < 0.05, ^§§^ *p* < 0.01, ^§§§^ *p* < 0.001 vs hDFs + TGFβ 3 days and hDFs + TGFβ 7 days; ^###^ *p* < 0.001 vs 3 days TGFβ^stim^ hDFs after hDPSC co-culture.

hDFs P3 were cultured under TGF-β1 stimulation for 3 and 7 days to mimic different times of exposure toward a pro-fibrotic microenvironment, and then cells were evaluated for the expression of pro-fibrotic markers, before and after culturing with hDPSCs for 4 days through a transwell co-culture system (Figure 1). Data from real-time PCR analyses showed that, after 3 days of stimulation, TGF-β1 induced an upregulation of the myofibroblast-associated genes, as revealed by a statistically significant increase in mRNA levels of ACTA2, HDAC4, and SPARC (**p < 0.01, ***p < 0.001 vs ctrl hDFs, Figure 1A). After a prolonged exposure to the pro-fibrotic stimulus (7 days), hDFs revealed a statistically significant increase in mRNA levels of ACTA2, COL1A1, FN1, HDAC4, SPARC, and TNC, when compared to the control group (*p < 0.05, **p < 0.01, ***p < 0.001 vs ctrl hDFs, Figure 1A) with COL1A1, FN1, and TNC being further increased in a statistically significant manner when compared to the 3-day stimulation group (°*p* < 0.05, °°*p* < 0.01, °°°*p* < 0.001 vs hDFs + TGF-β 3 days, Figure 1A), which demonstrated the ability of TGF-β1 to induce a fibroblast-to-myofibroblast conversion.

After transwell co-culture with hDPSCs, hDFs pre-stimulated with TGF-β1 for 3 and 7 days showed a downregulation of all the pro-fibrotic genes, except for COL1A1 and TNC, which showed statistically significant decreased mRNA levels only after 7 days of pre-stimulation with TGF-β1 (^§^ *p* < 0.05, ^§§^ *p* < 0.01, ^§§§^ *p* < 0.001 vs hDFs + TGF-β 3 days and hDFs + TGFβ 7 days, Figure 1A).

Western blot analyses further confirmed the ability of TGF-β1 to induce statistically significant increased expression levels of α-SMA and fibronectin after either 3 or 7 days of stimulation (**p < 0.01, ***p < 0.001 vs ctrl hDFs, Figure 1B), with a further statistically significant increase in expression of both markers in the 7-day group, when compared to the 3-day group (°°°*p* < 0.001 vs hDFs + TGFβ 3 days), showing that a longer stimulation induced a stronger commitment toward the myofibroblast phenotype (Figure 1B). After co-culturing with hDPSCs through the transwell system, hDFs pre-stimulated with TGF-β1 for 3 days showed, at the protein level, a statistically significant increased expression of α-SMA with respect to the experimental counterpart hDFs cultured alone (^§§^ *p* < 0.01 vs hDFs + TGFβ 3 days), whereas the opposite trend was revealed in 7-day TGF-β1^stim^ hDFs after co-culture with hDPSCs (^§^ *p* < 0.05 vs hDFs + TGFβ 7 days). Similar data were obtained by WB analysis of fibronectin in the same experimental groups (^§§§^ *p* < 0.001 vs hDFs + TGFβ 3 days and vs hDFs + TGFβ 7 days). Moreover, after co-culture with hDPSCs, the hDFs pre-stimulated with TGF-β1 for 7 days showed statistically significant lower levels of fibronectin, when compared to the counterpart hDFs pre-stimulated with TGF-β1 for 3 days (^###^ *p* < 0.001 vs 3-day TGFβ^stim^ hDFs after hDPSC co-culture).

**FIGURE 2:**
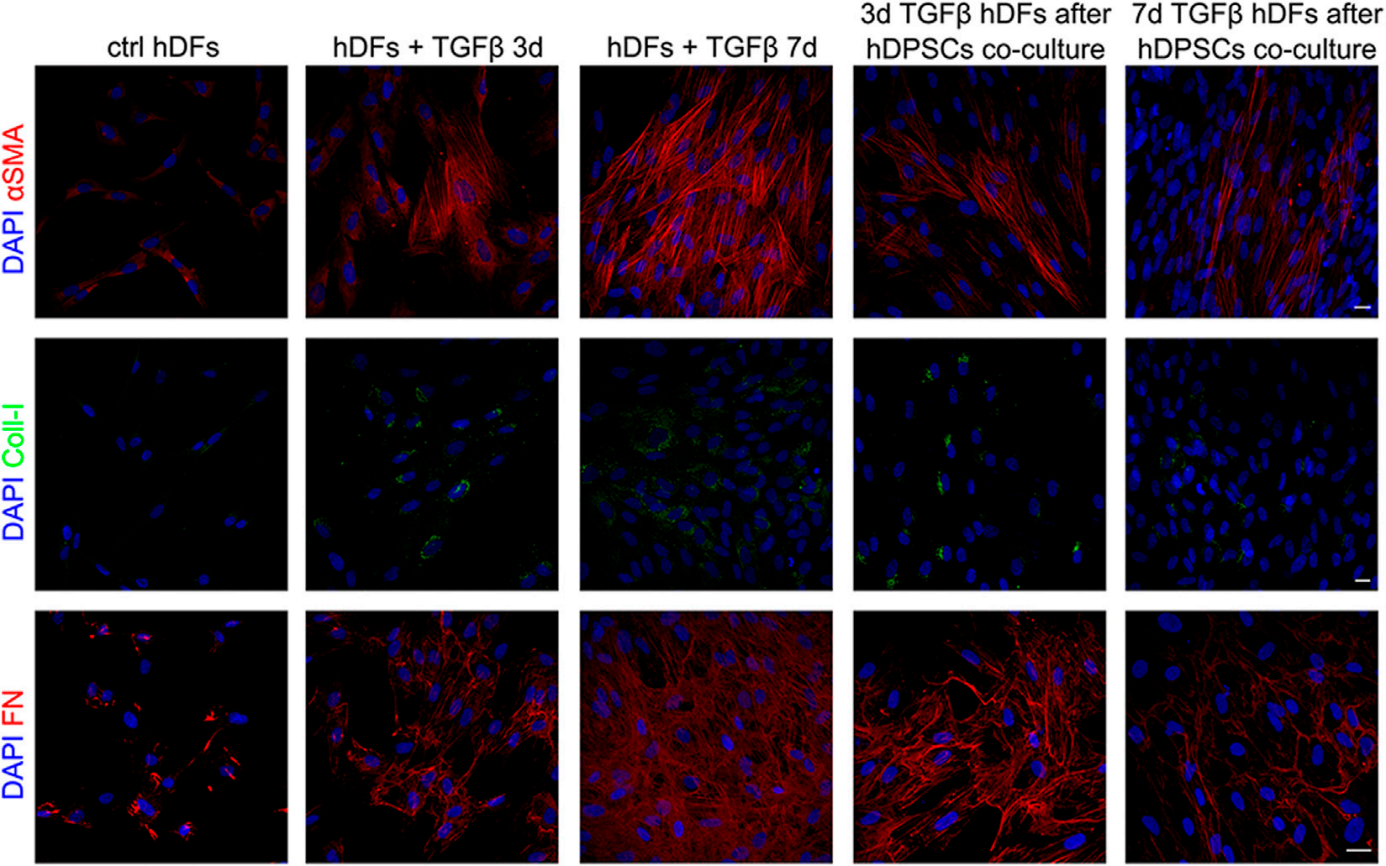
Confocal immunofluorescence analyses of pro-fibrotic markers in hDFs pre-stimulated with TGF-β1 cultured alone and after co-culture with hDPSCs. Representative immunofluorescence images showing the expression of α-SMA, collagen I, and fibronectin in hDFs (P3) after TGF-β1 stimulation and after co-culture with hDPSCs. Unstimulated hDFs were used as controls. Nuclei were counterstained with DAPI. Scale bar: 10 μm.

Confocal immunofluorescence analyses further proved that the fibroblast-to-myofibroblast conversion was successfully triggered by TGF-β1 and that co-culture with hDPSCs was able to induce a reduction in the myofibroblast-associated markers α-SMA, Coll-I, and fibronectin (Figure 2). In particular, a consistent remodeling of the ECM-associated component fibronectin is shown in Figure 2, besides a morphological shift/cytoskeletal rearrangement linking to a decreased expression of α-SMA. These data suggest that hDPSCs are able to modulate the phenotype of early culture passage fibroblasts, after short and prolonged times of pro-fibrotic stimulations.

**FIGURE 3:**
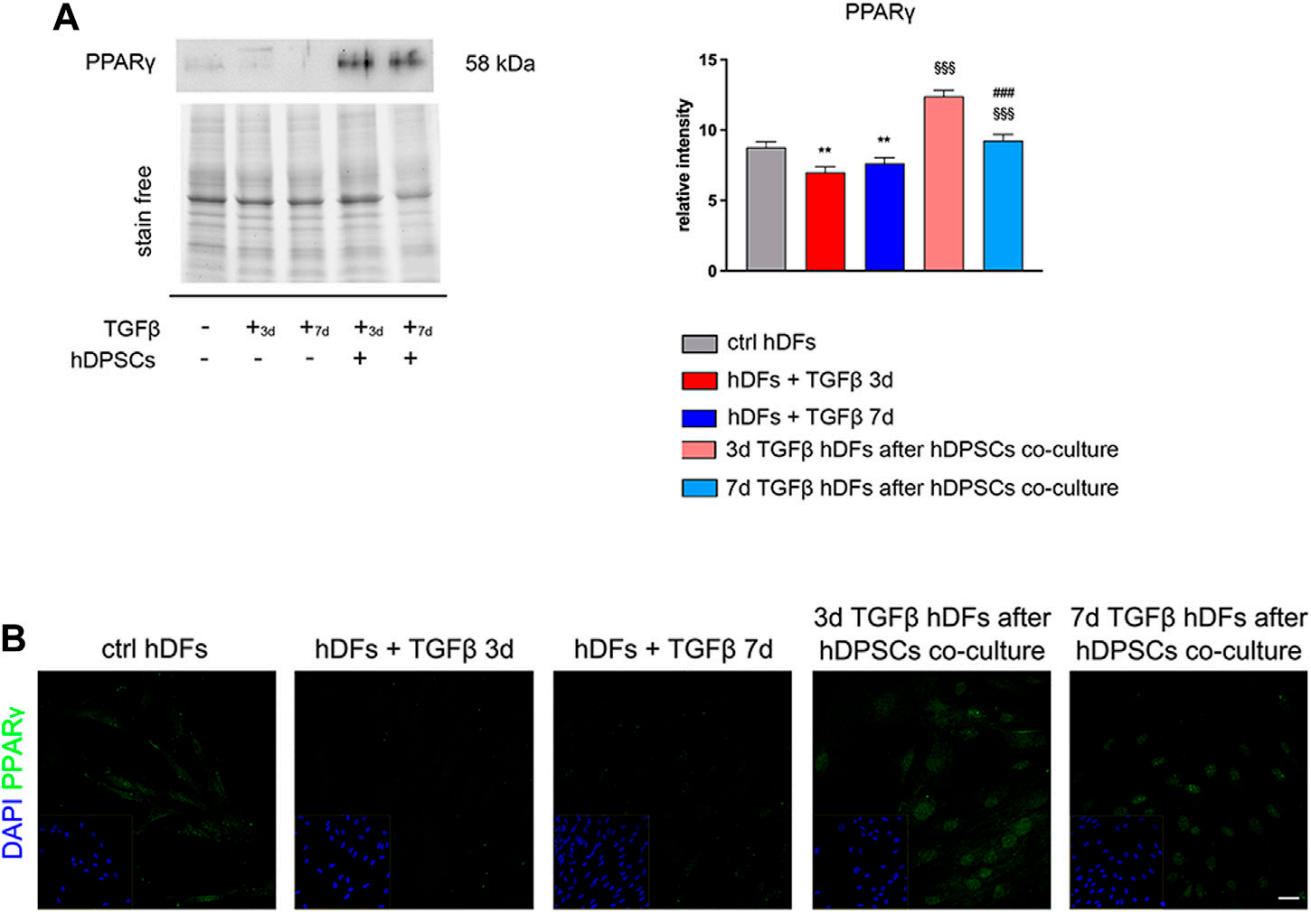
hDPSC co-culture can modulate PPARγ expression in hDFs in a pro-fibrotic microenvironment. **(A)** Western blot analysis of PPARγ was performed on hDFs (P3) pre-stimulated with TGF-β1, for 3 and 7 days, alone and after hDPSC co-culture. Histograms show that PPARγ expression was decreased in a statistically significant manner when hDFs were exposed to TGF-β1 and that, on the contrary, hDPSCs co-culture induced a statistically significantly increase in PPARγ. Data are expressed as mean ± SD and analyzed by one-way ANOVA followed by the Newman–Keuls *post hoc* test. **p < 0.01 vs ctrl hDFs; ^§§§^ *p* < 0.001 vs hDFs + TGFβ 3 days and hDFs + TGFβ 7 days. **(B)** Confocal immunofluorescence analysis of PPARγ expression among different hDF experimental groups. Nuclei were counterstained with DAPI and are reported in yellow squared inserts. Scale bar: 10 μm.

Peroxisome proliferator-activated receptor γ (PPARγ) has been demonstrated to play important roles in regulating processes related to fibrogenesis—including cellular differentiation, inflammation, and wound healing—by counteracting TGF-β1 pro-fibrotic effects (Uto et al., 2005; Lakatos et al., 2007). Notably, as shown in Figure 3, Western blot analysis highlighted that TGF-β1 induced a decreased expression of PPARγ in hDFs, and that it was instead increased in a statistically significant manner, in both experimental groups of TGF-β1-pre-stimulated hDFs after co-culture with hDPSCs (^§§§^ *p* < 0.001 vs hDFs + TGFβ 3 days and vs hDFs + TGFβ 7 days; Figure 3A).

In particular, a strong nuclear immunolabeling against PPARγ was observed in TGF-β-pre-stimulated hDFs after co-culturing with hDPSCs, hinting the activation of anti-fibrotic mechanisms contrasting the fibrogenic outcome of TGF-β1 stimulation (Figure 3B).

### 3.2 Effects of the pro-fibrotic microenvironment on hDPSCs

**FIGURE 4:**
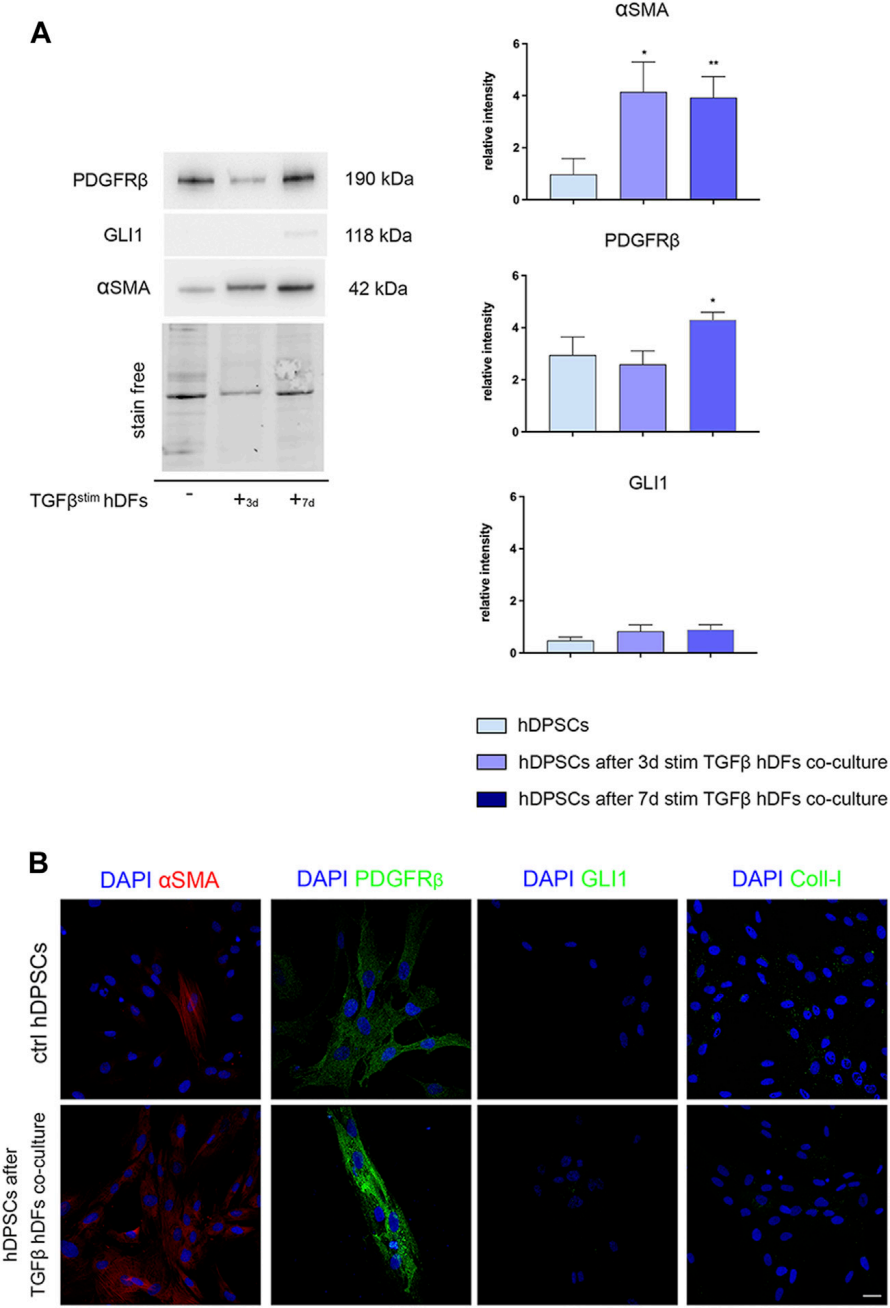
Evaluation of the hDPSC phenotype after exposure to the early pro-fibrotic microenvironment. The expression of PDGFRβ, GLI1, α-SMA, and Coll-I was evaluated by Western blot **(A)** and confocal immunofluorescence analyses **(B)**, in hDPSCs after co-culturing with TGF-β1-pre-stimulated hDFs under pro-fibrotic conditions. **(A)** Histograms show the relative intensity of PDGFRβ, GLI1, and α-SMA. Data are presented as mean ± SD and analyzed by one-way ANOVA followed by Dunnett’s *post hoc* test. *p < 0.05, **p < 0.01 vs ctrl hDPSCs. **(B)** Immunofluorescence analysis of α-SMA, PDGFRβ, GLI1, and Coll-I in hDPSCs after co-culture with TGF-β-pre-stimulated hDFs. hDPSCs cultured alone were used as controls. Nuclei were counterstained with DAPI. Scale bar: 20 μm.

hDPSCs were co-cultured through a transwell system with hDFs pre-stimulated by TGF-β1 for 3 and 7 days, still in the presence of TGF-β1, to mimic a fibro-inflammatory microenvironment *in vitro*. As shown in Figure 4, Western blot analyses revealed that a statistically significant increased expression of α-SMA was induced in hDPSCs under these co-culture conditions in both the experimental groups, when compared to control hDPSCs cultured alone (*p < 0.05, **p < 0.01 vs. hDPSCs, Figure 4A), whereas no statistically significant difference was detected among the two co-culture groups.

In parallel, PDGFRβ expression was statistically significantly higher in hDPSCs after co-culture with hDFs pre-stimulated with TGF-β1 for 7 days (*p < 0.05, vs hDPSCs), whereas no statistically significant difference was detected in GLI1 expression among the three experimental groups (Figure 4A). Confocal immunofluorescence analyses further confirmed these findings, besides revealing no immunolabeling against GLI1 and Coll-I (Figure 4B). These data highlight that after culturing under pro-fibrotic conditions, hDPSCs enhanced their pericyte-like features, without being induced toward a pro-fibrotic/myofibroblast phenotype.

### 3.3 BMP2 modulation in hDPSCs and its effects on the TGF-β1-induced pro-fibrotic phenotype in hDFs

**FIGURE 5:**
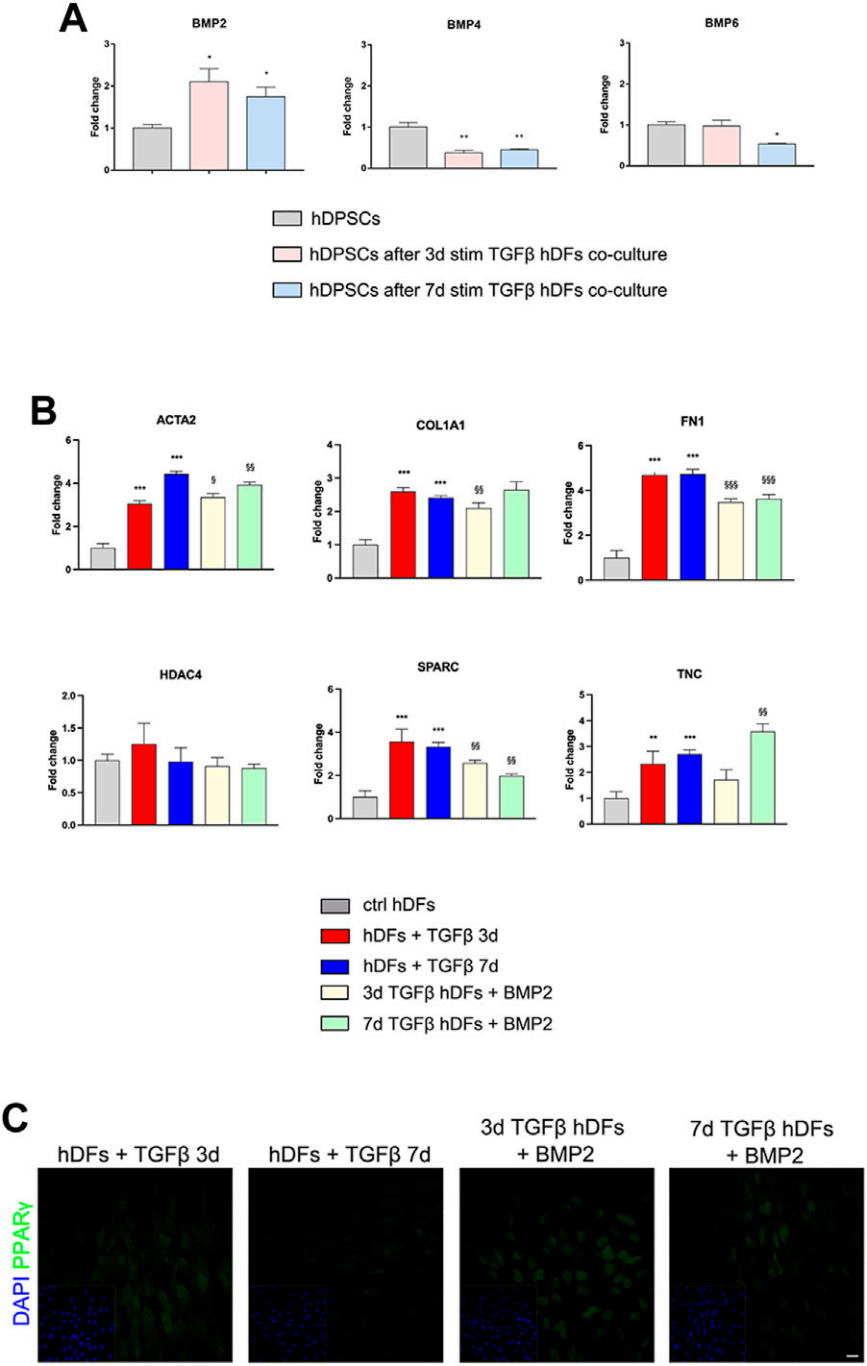
Involvement of BMPs in the modulation of the pro-fibrotic commitment of hDFs by hDPSCs. **(A)** Real-time PCR analysis of BMP2, BMP4, and BMP6 was performed in hDPSCs after co-culture with TGF-β1-pre-stimulated hDFs (P3). Histograms show a statistically significantly increase in BMP2 mRNA levels in hDPSCs after co-culture with hDFs pre-stimulated with TGF-β1 either for 3 or 7 days. Data are expressed as mean ± SD and analyzed by one-way ANOVA followed by Dunnett’s *post hoc* test. *p < 0.05, **p < 0.01 vs ctrl hDPSCs. **(B)** Real-time PCR analyses of pro-fibrotic markers were carried out in TGF-β1-pre-stimulated hDFs after exposure to 50 ng/mL human recombinant BMP2. Histograms show that BMP2 treatment induced a modulation of the mRNA levels of pro-fibrotic markers in hDFs that is similar to hDPSC co-culture. Data are expressed as mean ± SD and analyzed by one-way ANOVA followed by the Newman–Keuls *post hoc* test. **p < 0.01, ***p < 0.001 vs ctrl hDFs; ^§^ *p* < 0.05, ^§§^ *p* < 0.01, ^§§§^ *p* < 0.001 vs hDFs + TGFβ 3 days and hDFs + TGFβ 7 days. **(C)** Representative confocal immunofluorescence images showing the increased expression of PPARγ expression in TGF-β1-pre-stimulated hDFs after BMP2 treatment. Nuclei were counterstained with DAPI and reported in yellow squared inserts. Scale bar: 10 μm.

The modulation of BMP2, BMP4, and BMP6 in hDPSCs was evaluated by real-time PCR analyses (Figure 5A). Histograms in Figure 5A show a statistically significant fold increase in BMP2 mRNA levels after co-culture with 3 and 7 days TGF-β1-pre-stimulated hDFs, when compared to control hDPSCs cultured alone (*p < 0.05 vs hDPSCs). At the same time, mRNA levels of BMP4 were decreased in hDPSCs after co-culture with hDFs under both pre-stimulation conditions (**p < 0.01 vs hDPSCs) with respect to the control group, whereas mRNA levels of BMP6 revealed a statistically significant fold decrease only in hDPSCs after co-culture with 7-day TGFβ^stim^ hDFs (*p < 0.05 vs hDPSCs; Figure 5A).

Based on these data, hDFs (P3) pre-stimulated with TGF-β1 for 3 and 7 days were cultured in the presence of rhBMP2 for 4 days; then, real-time PCR analyses were carried out on hDFs from each experimental group to evaluate the effects of rhBMP2 on the expression of myofibroblast-associated genes ACTA2, COL1A1, FN1, HDAC4, SPARC, and TNC. Figure 5B shows that a statistically significant reduction in mRNA levels of COL1A1, FN1, and SPARC was observed in 3-day TGF-β1-pre-stimulated hDFs + BMP2 (^§§^ *p* < 0.01, ^§§§^ *p* < 0.001 vs hDFs + TGFβ 3 days), whereas only ACTA2, FN1, and SPARC showed a statistically significant decrease in mRNA levels in 7-day TGF-β1-pre-stimulated hDFs + BMP2 (^§§^ *p* < 0.01, ^§§§^ *p* < 0.001 vs hDFs + TGFβ 7 days).

Moreover, confocal immunofluorescence analyses revealed that immunolabeling against PPARγ was stronger and specifically localized at the nuclear level in TGF-β1-pre-stimulated hDFs exposed to rhBMP2, suggesting that this stimulus induced a myofibroblast-to-lipofibroblast conversion (Figure 5C).

These data may support the hypothesis that BMP2 pathway activation is one of the mechanisms leveraged by hDPSCs to exert an anti-fibrotic action in modulating the myofibroblast phenotype induced by TGF-β1 stimulation.

### 3.4 Effects of pro-fibrotic stimulation on co-cultures between hDPSCs and late culture passage fibroblasts

**FIGURE 6:**
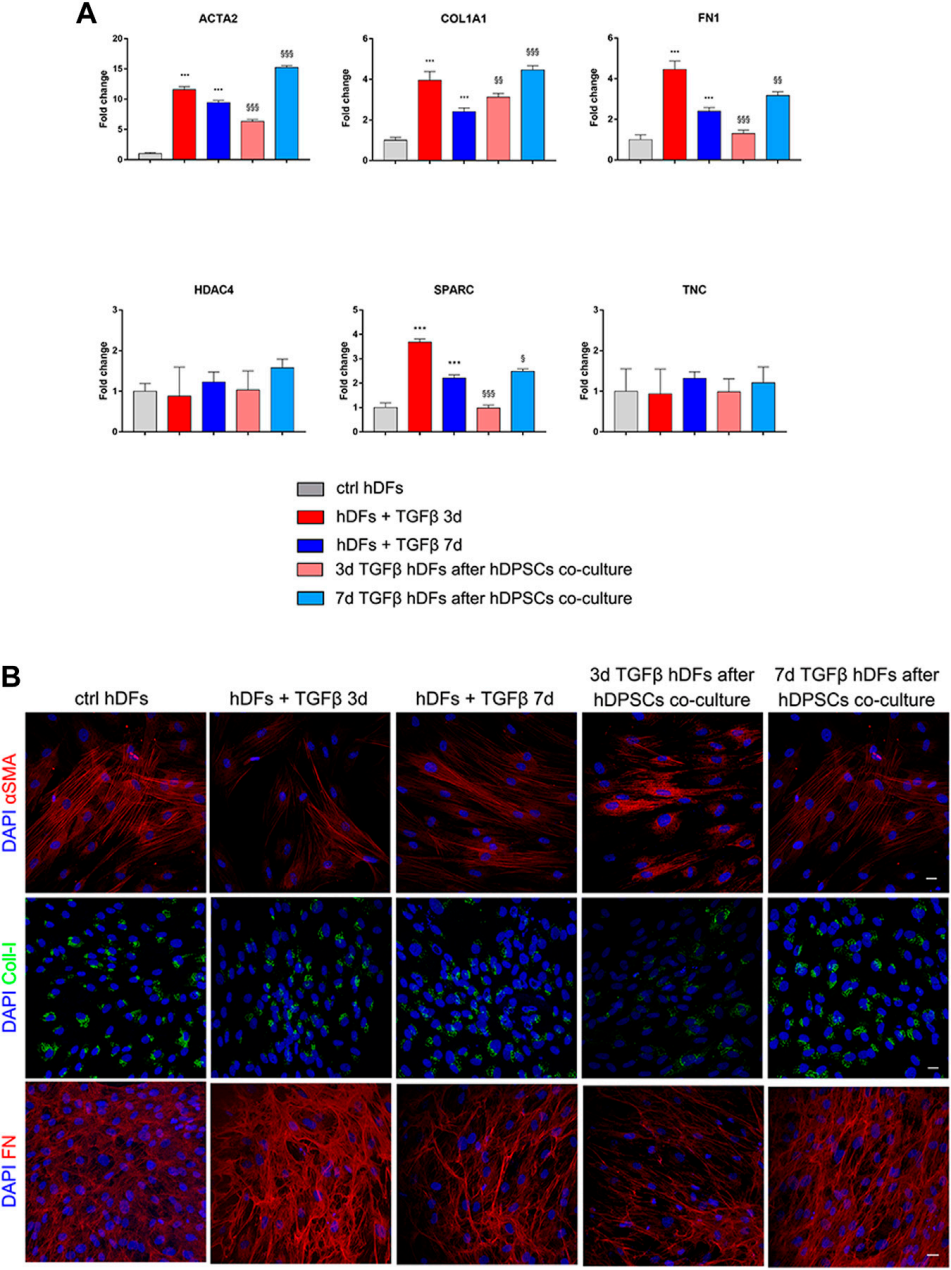
Effects of the pro-fibrotic microenvironment on late passage hDFs. **(A)** The expression of ACTA2, COL1A1, FN1, HDAC4, SPARC, and TNC was evaluated through real-time PCR analyses of hDFs (P12) stimulated with TGF-β1, cultured alone and after co-culture with hDPSCs. Data are expressed as mean ± SD and analyzed by one-way ANOVA followed by the Newman–Keuls *post hoc* test. ***p < 0.001 vs ctrl hDFs; ^§^ *p* < 0.05, ^§§^ *p* < 0.01, ^§§§^ *p* < 0.001 vs hDFs + TGFβ 3 days and hDFs + TGFβ 7 days. **(B)** Representative confocal immunofluorescence images showing the expression of α-SMA, collagen I, and fibronectin in late passage hDFs after TGF-β1 stimulation and after co-culture with hDPSCs. Unstimulated hDFs (P12) were used as controls. Nuclei were counterstained with DAPI. Scale bar: 10 μm.

hDFs at the late culture passage (P12) were cultured under the TGF-β1 stimulation for 3 and 7 days, to mimic pro-fibrotic stimulation on fibroblasts that are more committed toward a myofibroblast phenotype, and then transwell co-culture systems were set up with hDPSCs, still in the presence of TGF-β1 for further 4 days. Data from real-time PCR analyses (Figure 6A) showed that after co-culture with hDPSCs, only 3-day TGF-β1-pre-stimulated fibroblasts reduced their mRNA levels of ACTA2, COL1A1, FN1, and SPARC in a statistically significant manner (^§§^ *p* < 0.01, ^§§§^ *p* < 0.001 vs hDFs + TGFβ 3 days). Conversely, in 7-day TGF-β1-pre-stimulated fibroblasts, the mRNA levels of expression of all these pro-fibrotic markers were statistically significantly increased after co-culture with hDPSCs, when compared to the counterpart cultured alone (^§^ *p* < 0.05, ^§§^ *p* < 0.01, ^§§§^ *p* < 0.001 vs hDFs + TGFβ 7 days).

**FIGURE 7:**
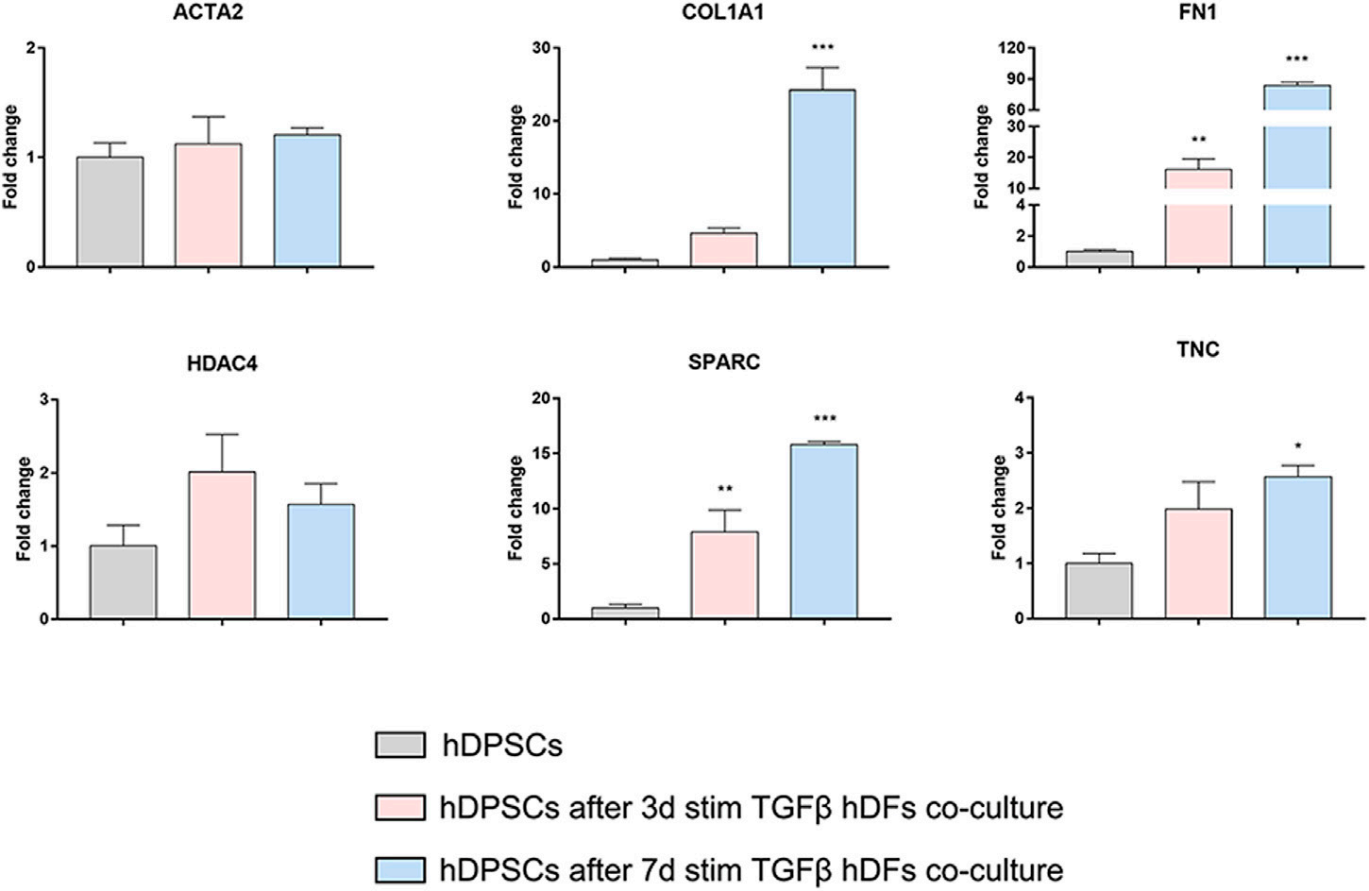
Effects of the pro-fibrotic microenvironment on hDPSCs after co-culture with late passage hDFs. Real-time PCR analyses showing the upregulation of pro-fibrotic markers in hDPSCs after co-culture with TGF-β1-pre-stimulated hDFs (P12). Histograms report a statistically significantly increased fold change in mRNA levels of COL1A1, FN1, SPARC, and TNC when compared to hDPSCs cultured alone. Data are expressed as mean ± SD and analyzed by one-way ANOVA followed by Dunnett’s *post hoc* test. *p < 0.05, **p < 0.01, ***p < 0.001 vs hDPSCs.

Confocal immunofluorescence analyses highlighted that hDFs (P12) already expressed α-SMA and the pro-fibrotic ECM markers, i.e., Coll-I and FN, whose expression was maintained after TGF-β1 stimulation. Furthermore, immunofluorescence analyses also clearly showed that hDPSCs co-culture was not able to induce a decrease in expression of such markers in late passage TGF-β1-pre-stimulated fibroblasts (Figure 6B). The effects of such a pro-fibrotic co-culture system were investigated in hDPSCs as well. Figure 7 shows that real-time PCR analyses revealed that the mRNA levels of fibrotic ECM-associated markers was statistically significantly higher in hDPSCs after co-culturing with TGF-β1-pre-stimulated fibroblasts P12, in the presence of TGF-β1 stimulation (**p < 0.01, hDPSCs after 3-day^stim^ TGFβ vs hDPSC ctrl; *p < 0.05, ***p < 0.001 hDPSCs after 7-day^stim^ TGFβ vs hDPSC ctrl).

## 4 Discussion

Human dental pulp stem cells reside in the perivascular area of the dental pulp and own peculiar stemness properties ranging from a wide differentiation potential, owing to their neural crest origin, to immunomodulatory properties that rely on the activation of different molecular pathways (Di Tinco et al., 2021). Moreover, their well-documented *in vivo* contribution to the regeneration of different tissue injuries is exerted through histointegration, cell differentiation, and paracrine effects that support the recovery of tissue homeostasis, i.e., the promotion of neoangiogenesis and the reduction of fibrosis (Carnevale et al., 2018; Luzuriaga et al., 2019; Merckx et al., 2020). This evidence is corroborated by findings from our research group. Particularly, we have observed that after direct contribution of hDPSCs to *in vivo* tissue regeneration, a reduction of fibrosis was appreciable in each experimental model of tissue injury (Pisciotta et al., 2015; Zordani et al., 2019). This contribution is in accordance with data reviewed by Qin et al. (2023), particularly, implantations of external MSCs are reported with effective and protective functions in the treatment of fibrotic diseases in both pre-clinical and clinical studies (Alfaifi et al., 2018; Peltzer et al., 2018; Li et al., 2021; Qin et al., 2023). Additionally, resident MSCs are reported to be responsible for fibrotic development in various tissues since they represent one major source of myofibroblasts when exposed to an inflammatory microenvironment. In this regard, the role of MSCs in fibro-inflammatory processes is controversial and widely debated because the myofibroblasts originate not only from MSCs but also from smooth muscle cells and pericytes, local resident fibroblasts, and epithelial or endothelial cells after epithelial-/endothelial-to-mesenchymal transition (Micallef et al., 2012). Hence, the aim of our study is to investigate the role of hDPSCs in an *in vitro* model of a fibro-inflammatory process. Various cytokines and growth factors play a role in the development of fibrotic processes. Among these, TGF-β is the main factor leading to fibrosis. The three TGF-β isoforms, i.e., TGF-β1, -β2, and -β3, are primary regulators of cell differentiation, migration, proliferation, and gene expression and have been implicated in both reparative and fibrotic responses. The role of TGF-βs in mediating tissue fibrosis is supported by several *in vitro* studies, animal model experiments, and clinical findings (Anscher et al., 1993; Sonnylal et al., 2007; Rice et al., 2015).

Moreover, although all three isoforms are expressed in fibrotic tissues, the development of tissue fibrosis is primarily attributed to TGF-β1 (Ask et al., 2008; Biernacka et al., 2011). Fibroblasts exposed to TGF-β1 are induced to express α-SMA, becoming activated myofibroblasts that initiate the deposition of large amounts of extracellular matrix components, such as type I collagen, fibronectin, and tenascin (Biernacka et al., 2011). However, TGF-β contributes to the pathogenesis of fibrosis by acting on different cell types, including perivascular cells (Frangogiannis, 2020).

In our study, we used hDFs at the early and late culture passages and exposed them to TGF-β1 for different experimental times. Interestingly, we demonstrated that, when TGF-β1 is added to hDFs at early culture passages, they are induced toward a fibroblast-to-myofibroblast conversion, as shown by the upregulation of α-SMA and the main biomarkers of fibrosis-related ECM remodeling, either at mRNA or protein levels, thus confirming that a fibrogenetic microenvironment was induced, which is in compliance with previous findings (Biernacka et al., 2011).

Notably, the establishment of a transwell co-culture system between early culture passage hDFs and hDPSCs in the presence of TGF-β1 induced a modulation of the myofibroblast phenotype, as confirmed by a significant downregulation of α-SMA and pro-fibrotic ECM-related markers. At the same time, under these co-culture conditions, we observed that PPARγ expression was upregulated in the early culture passage hDFs and, as supported by confocal immunofluorescence analyses, nuclear translocation occurred, suggesting a myofibroblast-to-lipofibroblast transition. These data are accompanied by an upregulated expression of BMP2 in hDPSCs under transwell co-culture conditions. As established in the literature, TGF-β1 and BMP2 pathways regulate processes acting as antagonists in the fibrogenesis process. As shown in previous studies and confirmed by our data, BMP2 has an anti-fibrotic effect that counteracts TGF-β1 (Wang et al., 2012; Chung et al., 2018). In particular, when human recombinant BMP2 was added to early hDFs pre-stimulated with TGF-β1, the myofibroblast-associated markers were downregulated. This evidence demonstrates that BMP2 plays a pivotal role in the modulation of fibrogenic processes. Moreover, data regarding hDPSCs showed that, when co-cultured with early culture passage hDFs in the presence of TGF-β1, they did not undergo conversion toward the myofibroblast phenotype, rather maintaining the expression of the pericyte-like cell markers.

hDFs at late culture passages already expressed α-SMA and ECM-related markers. When exposed to TGF-β1 at different experimental times, the pro-fibrotic markers were further upregulated, except for HDAC4 and TNC. The establishment of co-culture with hDPSCs in the presence of TGF-β1 partially downregulated mRNA levels of ACTA2, FN1, and SPARC only at 3 days of stimulation, whereas no difference was observed in the 7-day stimulation group. At the same experimental times, analyses performed on hDPSCs revealed that they themselves increased their expression of pro-fibrotic markers.

Taken together, these data suggest that hDPSCs are able to modulate the pro-fibrotic microenvironment by preventing the conversion of fibroblast to myofibroblasts and stimulating a switch toward the lipofibroblast phenotype, through the activation of BMP2- and PPARγ-mediated molecular mechanisms, in accordance with previous findings (Calvier et al., 2017). In this regard, recent evidence from studies on animal models of liver and lung fibrosis has highlighted that activated myofibroblasts may undergo a dedifferentiation process by switching toward a quiescent lipofibroblast phenotype when fibrosis resolution occurs, through the activation of PPARγ signaling (Kisseleva et al., 2012; El Agha et al., 2017b; Kheirollahi et al., 2019). Based on the literature and our present findings, we argued that BMP2 upregulation in hDPSCs might have exerted either an autocrine effect on stem cells themselves, avoiding their activation toward myofibroblasts, or paracrine effects on TGF-β1-pre-stimulated hDFs, leading their transition toward the lipofibroblast phenotype.

At the same time, when a pro-fibrotic compartment is already activated and further induced toward the deposition of ECM components, hDPSCs are no longer capable of exerting a modulatory effect; in fact, although further investigations are needed to corroborate this hypothesis, the risk is that stem cells acting as external contributors might exacerbate the evolution of pro-fibrotic processes by transforming themselves into main actors of fibro-inflammatory processes.

## Data Availability

The original contributions presented in the study are included in the article; further inquiries can be directed to the corresponding author.
